# Diagnostic completeness: the missing link in antimicrobial stewardship in low- and middle-income countries

**DOI:** 10.7189/jogh.16.03020

**Published:** 2026-08-28

**Authors:** Qingchun Pan, Xiaohua Chen

**Affiliations:** Department of Infectious Diseases, Shanghai Sixth People’s Hospital Affiliated to Shanghai Jiao Tong University School of Medicine, Shanghai, China

**Keywords:** antimicrobial resistance, diagnostic completeness, antimicrobial stewardship, primary care, point-of-care testing, LMICs

## Abstract

Antimicrobial resistance (AMR) is accelerating in low- and middle-income countries, yet antibiotic prescribing decisions are often made without diagnostic certainty. Diagnostic completeness – the systematic commitment to obtaining a confirmed diagnosis before initiating treatment – is an overlooked upstream component of antimicrobial stewardship. Without this pre-treatment commitment, downstream antimicrobial management efforts risk being bypassed because diagnostic testing is never ordered. Here, we distinguish diagnostic completeness from diagnostic stewardship: the former concerns whether a diagnosis is pursued before treatment, whereas the latter concerns whether the appropriate diagnostic test is used. Behavioural alerts, hub-and-spoke diagnostics, and incentive redesign are three practical pathways that could strengthen diagnostic completeness in primary care, although each faces well-documented implementation barriers requiring context-specific adaptation. We also address patient-level constraints, equity implications, health system heterogeneity, and empirical therapy in emergency contexts. Diagnostic completeness is a necessity; closing the diagnostic gap will not solve AMR alone, yet without it, stewardship will not suffice.

Antimicrobial resistance (AMR) claimed 1.27 million lives in 2019, with low- and middle-income countries (LMICs) bearing a disproportionate burden [1]. The 2025 World Health Organization (WHO) Global Antimicrobial Resistance Surveillance Report found that one in six bacterial infections globally is now resistant to common antibiotics, with resistance rising annually by 5–15% in over 40% of pathogen–antibiotic combinations [2]. International health organisations have promoted antimicrobial stewardship programmes (ASPs) as a cornerstone of AMR containment. Yet after nearly two decades, a fundamental reality remains unaddressed: many antibiotic prescriptions in LMICs are written without a confirmed aetiological diagnosis [3].

Securing a diagnosis before treatment is a core principle of evidence-based medicine and has been implicit in stewardship. However, what remains missing is a systematic framework that positions this pre-treatment commitment at the centre of antimicrobial stewardship as a prerequisite, not as an adjunct. A recent study called for health system strengthening in LMICs to combat AMR, highlighting regulation, primary care stewardship, and digital health tools, while also identifying expanded access to basic diagnostics as an important priority [4].

We build upon this foundation in two ways. First, diagnostic completeness requires explicit articulation, systematisation, and operationalisation as a measurable construct for LMIC settings. Without this upstream commitment, downstream stewardship efforts risk being undermined entirely because testing is never ordered. Therefore, effective stewardship depends on diagnostic certainty, as antimicrobial use can only be appropriately managed when the underlying condition has been adequately identified. Second, while Sorn [4] focuses on macro-level reforms, we present three front-line pathways: behavioural alerts, hub-and-spoke diagnostics, and incentive redesign, each of which faces well-documented implementation barriers. This advocacy piece calls for more systematic implementation of established principles.

## DEFINING DIAGNOSTIC COMPLETENESS

Diagnostic completeness means systematically securing a confirmed diagnosis before starting therapy, whenever clinically feasible, translating WHO patient safety principles into frontline practice in resource-limited settings. In practice, this means prioritising diagnostic evaluation over rapid clinical decisions; avoiding drug treatments that obscure clinical signs before a diagnostic workup is completed; and ensuring that when diagnoses are missed, accountability lies with the system rather than the individual clinician.

Diagnostic completeness is an overlooked upstream dimension of WHO’s diagnostic stewardship, which asks ‘Are we using the right test?’, whereas completeness asks ‘Have we really committed to a confirmed diagnosis before treating?’ In this sense, the former is the logical prerequisite, while the latter provides the framework for optimisation. (**Table 1**). Both exist along a continuum, with diagnostic completeness representing the pre-analytical commitment that logically precedes the analytical focus of diagnostic stewardship. Therefore, the distinction is one of sequence and emphasis rather than a difference in nature.

**Table 1:** Comparison between diagnostic completeness and diagnostic stewardship

Dimension	Diagnostic completeness	Diagnostic stewardship
Core question	Have we committed to obtaining a confirmed diagnosis before treatment?	Are we using the right test for the right patient?
Prerequisite	The decision to test must be made first	Testing is already deemed necessary
Focus of action	Ensuring diagnostic evaluation precedes empirical treatment	Optimising test selection, timing, and interpretation
Behaviour prevented	Empirical prescribing that bypasses diagnostic testing	Unnecessary, inappropriate, or excessive testing
Consequence of failure	Diagnosis bypassed → aetiology unknown → inappropriate treatment continues	Tests misused → results misinterpreted → suboptimal treatment guided
Key indicator	Proportion of febrile patients receiving a confirmed diagnosis before antibiotic prescription	Test positivity rate, antibiotic prescribing rate, guideline adherence
Relation to AMR	Upstream prevention: reduces empirical antibiotic use	Midstream optimisation: ensures test results guide therapy

AMR – antimicrobial resistance

Terms such as ‘improving diagnostic access’ or ‘timely diagnosis’ could mean getting any test quickly. Those phrases do not capture the precondition that diagnosis must be secured before treatment begins. In LMICs, a common failure is that testing is never ordered and treatment begins empirically [3]. Diagnostic completeness fills this gap by articulating the pre-analytical commitment that should precede both testing and treatment.

## THE SCALE OF DIAGNOSTIC NEGLECT AND THE CASE FOR INTERVENTION

The Lancet Commission on Diagnostics estimated that 47% of the global population lacks access to diagnostics [5]. In LMICs, fewer than one in five people have access to even the simplest diagnostic tests [5]. In rural Tanzania and Uganda, basic microbiology services are available in fewer than 10% of primary health facilities, leaving clinicians with little choice but to treat empirically [3]. Prasad *et al.* found that over 80% of febrile cases had no identified cause [6], and Modgil *et al.* highlight that even where diagnostic tools exist, access remains inequitable, with marginalised populations disproportionately excluded from testing [7]. This ‘diagnostic void’ drives clinicians to broad-spectrum coverage when uncertain.

These deficits reflect deeper health system weaknesses, including regulatory enforcement, underfunded laboratory infrastructure, and limited stewardship capacity. Addressing AMR in LMICs therefore requires not merely expanding access to diagnostics, but redesigning the systems in which diagnostic decisions are made.

Gres *et al.* found the combination of ASP and point-of-care testing (POCT) had higher success rates (85%) than ASPs (73%) or POCT alone (80%), and a meta-analysis of clinical decision support systems (CDSS) showed a >80% reduction in antibiotic prescribing (odds ratio = 0.17; 95% confidence interval = 0.07–0.45) [8]. Despite high heterogeneity (*I*^2^ = 99.7%) in the CDSS meta-analysis, the direction of effect was consistent across studies. Kuitunen *et al.* found that rapid point-of-care respiratory pathogen testing, when implemented without accompanying stewardship interventions, did not reduce antibiotic prescribing [9], reinforcing that diagnostic tools are necessary but not sufficient.

The existing literature has primarily focused on intermediate outcomes, such as prescribing rates, guideline adherence, and test utilisation. Direct evidence that improving diagnostic completeness reduces AMR-related morbidity or mortality in LMIC primary care remains limited.

## OPERATIONALISING DIAGNOSTIC COMPLETENESS: TOWARD MEASURABLE INDICATORS

If diagnostic completeness is to move from principle to practice, it requires a measurable construct. We propose three candidate indicators for LMIC primary care: diagnostic completion rate, defined as the proportion of febrile patients with a confirmed aetiological diagnosis documented before antibiotic prescription; empirical prescription with documented rationale, which is the proportion of antibiotic prescriptions with a documented rationale when no confirmed diagnosis was obtained; and diagnostic testing rate before antibiotics, defined as the proportion of patients receiving a diagnostic test before or at the time of antibiotic prescription.

Furthermore, minimum data infrastructure includes a functioning health information system that documents diagnoses, prescriptions, and test orders at the individual level; standardised documentation protocols distinguishing confirmed from presumptive diagnoses; and regular data quality audits. In many LMIC primary care settings, electronic health records are not available, but paper-based registers could provide a feasible approach for tracking diagnostic processes. Also, in facilities without established data systems, readiness scores based on staffing, guidelines, equipment, diagnostics, and medicines could provide a simple way to assess aspects of diagnostic completeness.

## THREE PRACTICAL PATHWAYS TO STRENGTHEN DIAGNOSTIC COMPLETENESS

Behavioural alerts, hub-and-spoke diagnostics, and incentive redesign are our three proposed pathways that could strengthen diagnostic completeness. None is a standalone solution, and each face well-documented barriers and requires context-specific adaptation.

Behavioural alerts can disrupt the ‘prescribe-first’ reflex. In the USA primary care setting, displaying commitment letters with clinician photographs and signatures in examination rooms reduced inappropriate antibiotic prescribing for acute respiratory infections by 19.7 percentage points [10]. More broadly, Xu *et al.*’s meta-analysis of 56 trials estimated that audit-and-feedback interventions were associated with reductions of 11% in prescribing volume, 23% in unnecessary initiations, and 17% in broad-spectrum use [11].

This evidence, however, comes from high- or middle-income settings with substantial digital health infrastructure, including electronic health records, reliable electricity, and engaged prescribers, not broadly present in LMIC primary care. Where these are absent, paper-based checklists and clinical decision support flowcharts offer a fallback.

Point-of-care diagnostics need systematic deployment where they are most needed. Tuberculosis (TB) diagnostic networks in Mozambique and Tanzania offer relevant experience, where hub-and-spoke GeneXpert and decentralised Truenat testing have been compared [12,13]. TB diagnostics require molecular testing, sample transport, and laboratory infrastructure – conditions analogous to expanding diagnostic capacity for febrile illnesses. Where laboratory infrastructure is absent, hub-and-spoke models can serve as transitional solutions. However, such models face profound cold–chain, transportation, and personnel challenges. In Uganda, 61% of community health centres lacked refrigeration for specimen transport, and 91% lacked telephone or mobile communication to support hub-and-spoke systems [14]. Evidence from Mozambique and Tanzania shows that decentralised point-of-care testing (*i.e.* Truenat) reduced patient out-of-pocket costs and improved early treatment initiation compared to hub-and-spoke referral models [12,13]. Therefore, hub-and-spoke is one transitional option among several, not an ideal solution.

Incentive redesign is a longer-term goal that requires careful consideration. In fee-for-service systems, prescribing is driven by financial incentives; restructuring payment systems in LMICs requires years of policy development, healthcare financing reform, and international donor involvement. A scoping review of provider payment reforms in African Commonwealth countries found that evidence on provider payment reforms in LMICs remains limited, fragmented, and largely descriptive [15]. In salary- or capitation-based systems, inappropriate prescribing is driven more by diagnostic uncertainty, patient pressure, and risk aversion than by financial incentives. While some LMICs, including Rwanda, Tanzania, and Nigeria, have piloted performance-based financing (PBF) for primary care [15], no PBF programme has specifically targeted diagnostic completeness. We suggest this approach for future pilots.

## PATIENT-LEVEL CONSTRAINTS AND EQUITY IMPLICATIONS

Diagnostic completeness frameworks that focus exclusively on clinician behaviour risk overlooking a fundamental reality: patients often arrive having already self-medicated with antibiotics obtained informally, or they attend expecting a prescription and may seek care elsewhere if one is not provided. In northern Tanzania, 41–60% of antibiotics were obtained without prescription [16]; studies in rural Uganda show reliance on informal drug shops, poor health literacy, and limited diagnostics drive inappropriate antibiotic use [17]. Clinicians cannot achieve diagnostic completeness independently of patient expectations, prior antibiotic exposure, and health literacy, which are beyond their control.

These frameworks should be designed as system-level interventions, not individual clinician-level mandates. This requires community engagement and health literacy programmes, training providers in diagnostic communication, recognition that self-medication and prior antibiotic exposure complicate diagnostic interpretation, and regulation of informal antibiotic sales.

Furthermore, requiring diagnostic confirmation before treatment carries a clear equity risk. The most marginalised populations that are least able to afford tests, least likely to access facilities with testing capability, and most vulnerable to treatment delays could be systematically disadvantaged. Spatial analyses have demonstrated disproportionate diagnostic service distribution due to urban-rural disparities [7]. However, this risk is not an argument against diagnostic completeness; it is an argument for investing in diagnostic access for the marginalised. Three mitigation strategies are essential: subsidies or waivers for diagnostic tests for the poorest populations; tiered diagnostic pathways – simple RDTs at primary level with referral for complex testing only when necessary; and community-based sample collection and transport to reduce access barriers for remote populations. Equity-focused monitoring is essential to ensure the framework does not inadvertently exacerbate existing disparities.

## HEALTH SYSTEM HETEROGENEITY AND CONTEXT-SPECIFIC ADAPTATION

LMICs' health systems vary substantially in health financing, regulatory environments, disease epidemiology, laboratory infrastructure, and cultural norms. Diagnostic completeness is a descriptive framework, and not a prescriptive protocol. Four areas require context-specific adaptation: health financing (fee-for-service *vs* salary or capitation systems shape prescribing incentives differently); regulatory environments (antibiotic sales restrictions vary); disease epidemiology (diagnostic needs differ across settings); and laboratory infrastructure (ranging from none to well-equipped district hospitals). To account for these adaptations without compromising conceptual quality, we propose a tiered approach: the core principles remain constant, but the specific pathways, indicators, and implementation strategies are tailored to each context [18].

## EMPIRICAL THERAPY IN EMERGENCY CONTEXTS

Diagnostic completeness does not demand rigid adherence to ‘no treatment without diagnosis’ in emergencies where immediate therapy is life-saving. Empirical antibiotic therapy is clinically appropriate and often life-saving in many LMIC contexts, including suspected bacterial meningitis, severe pneumonia, sepsis, and febrile illness in malaria-endemic areas where diagnosis cannot safely precede treatment. We therefore distinguish between routine and emergency clinical contexts. In life-threatening conditions, diagnostic completeness is expressed as: start empirical treatment immediately, but collect diagnostic specimens simultaneously, and review and adapt therapy once results return. This is ‘diagnosis-enabled empirical therapy’ – not diagnosis-before-therapy, but diagnosis-parallel-to-therapy – ensuring that diagnostic completeness remains a guiding principle without compromising timely emergency care.

## IMPLEMENTATION: GOVERNANCE AND ACCOUNTABILITY

Our proposed pathways require significant investment, political commitment, and institutional capacity. In most LMIC health systems, responsibility is fragmented across ministries, regulatory bodies, professional associations, and facility-level management. Diagnostic completeness requires coordinated action across these levels – national health ministries could embed diagnostic completion indicators into routine health information systems; district health management teams could oversee data quality and feedback; facility-level clinical leaders could integrate diagnostic checklists into routine workflows. Costs vary enormously across settings, but upfront investments in transportation, cold chain, laboratory staffing, and communication infrastructure are substantial. Accountability requires mechanisms that hold systems accountable, not individuals: routine monitoring of diagnostic completion indicators; integration into quality assurance frameworks; regular feedback to clinicians; and transparent reporting to communities and policymakers.

## LIMITATIONS

First, the evidence linking diagnostic completeness to AMR-related morbidity and mortality is limited; most available evidence focuses on intermediate outcomes. Modelling studies indicate that molecular diagnostic testing may enable faster targeting of antibiotic therapies, with greatest impact at higher diagnostic coverage and shorter turnaround times [19], but prospective studies assessing long-term clinical outcomes remain a priority. Second, the pathways we propose face well-documented implementation barriers that are not fully addressed by the existing evidence. Third, the framework assumes health systems have the capacity to implement and monitor diagnostic completeness indicators; where health information systems are weak, this capacity must be built in parallel. Context-specific adaptation is essential, and the framework should be tested through prospective pilots before widespread use.

## CONCLUSIONS

The persistent failure to address diagnostic gaps in LMICs has left antimicrobial stewardship without its essential prerequisite – diagnosis itself. The evidence is clear: diagnostic support can reduce inappropriate prescribing. The gap in diagnostics is not a theoretical concern; it is a measurable reality. The framework we propose is an attempt to address that reality directly. Here, we articulate and systematise diagnostic completeness as a foundational principle for all such efforts. Where Sorn [4] provides a macro-structural agenda, we provide the micro-clinical translation – actionable at the point of care, using interventions that, while facing implementation barriers, represent promising directions for systematic piloting and adaptation. The principle of securing a diagnosis before treatment is not new. Our contribution is to give this principle a name, a measurable form, a set of concrete pathways, and a clear position in the sequence of stewardship – before testing, not after. You cannot steward what you cannot diagnose, and diagnostic completeness is a necessity. Policy-makers should start embedding diagnostic indicators into health system performance frameworks; clinicians should prioritise diagnostic evaluation before prescribing when feasible; and researchers should generate evidence on implementation and long-term outcomes. As Sorn [4] argued, it is time to act, and we add that it is time to act diagnostically.

## Data Availability

**Data availability:** No primary data were collected or analysed for this viewpoint. All cited evidence is derived from previously published studies as referenced, and no additional data were generated. The manuscript does not require data deposition in a repository.
